# Potential antimicrobial effect of plant essential oils and virulence genes expression in methicillin-resistant *Staphylococcus aureus* isolates

**DOI:** 10.14202/vetworld.2020.669-675

**Published:** 2020-04-13

**Authors:** Mohammad H. Gharaibeh, Mohammad S. Khalifeh, Esam M. Zattout, Luay F. Abu-Qatouseh

**Affiliations:** 1 Department of Basic Veterinary Medical Science, Faculty of Veterinary Medicine, Jordan University of Science and Technology, P.O. Box 3030 Irbid 22110 Jordan; 2 Department of Pharmacology and Biomedical Sciences, Faculty of Pharmacy, University of Petra, Amman, Jordan

**Keywords:** accessory gene regulator locus, antibacterial activity, essential oils, intercellular adhesion cluster, methicillin-resistant *Staphylococcus aureus*, minimum inhibitory concentration, virulence factors

## Abstract

**Aim:**

This study aimed to investigate the antibacterial efficacy of eight commercially available essential oil (EO) blends and characterize the effect on the expression of some virulence genes against methicillin-resistant *Staphylococcus aureus* (MRSA).

**Materials and Methods:**

*In vitro* evaluation of the antimicrobial effects of oils against MRSA was performed using the disk diffusion method and by measuring the minimum inhibitory concentration (MIC) and the minimum bactericidal concentration (MBC). The EOs (A-F) were contained (β-pinene, carvacrol, carvone, dimethyl trisulfide, linalool, limonene, menthol, monoterpene hydrocarbons, and thymol) in different amounts. In addition, a real-time polymerase chain reaction was also used to determine the gene expression of the virulence genes (intercellular adhesion cluster [ica]-9, ica-15, and RNA III) against MRSA (ATCC 43300) after treatment with selected oils.

**Results:**

Among the eight EOs evaluated, EO (D), (E), and (A) showed, in general, the greatest antimicrobial activity against MRSA. EO at 1/3 MIC has effectively down-regulated ica-9 and ica-15 of MRSA by 17.83 and 4.94 folds, respectively. Meanwhile, EO (A) has effectively down-regulated RNAIII by 3.74 folds. Our results indicated that some of the EOs exhibit promising antimicrobial effects against MRSA isolates. Moreover, the results of the analyzed virulence genes related to the pathogenicity of MRSA were down-regulated at the sub-MIC concentrations of EOs, indicated that EOs could be successfully used to suppress the virulence factors and, consequently, decreased the pathogenicity of MRSA.

**Conclusion:**

These encouraging results indicate that some of the EOs used in this study can be utilized as a natural antibiotic for the treatment of MRSA disease.

## Introduction

*Staphylococcus aureus* is considered as the main pathogen among Gram-positive bacteria that cause nosocomial infections [1]. The first case of clinical methicillin-resistant *S. aureus* (MRSA) was reported 58 years ago [2]. *S. aureus* has become resistant to many commonly used antibiotics due to the improper use of antibiotics as well as to the genetic plasticity of MRSA [3]. The development of antibiotic resistance has started with penicillin; this resistance was overcome with the use of penicillinase-stable methicillin. Later on, the widespread use of methicillin and their derivatives (such as methicillin, oxacillin, cloxacillin, and flucloxacillin) has led to the re-emergence of MRSA, which is very difficult to treat [4]. The ability of *S. aureus* to cause a variety of serious infections in humans and animals is linked to the expression of a group of factors that cooperate in the pathogenesis of infection in humans or animals. These expression factors on the cell surface of *S. aureus* play an important role in its virulence. *S. aureus* attaching to the cell surface of the host cells is mediated by many adhesion factors. For example, the intercellular adhesion cluster (ica) ADBC operon plays an important role in biofilm formation [5]. On the other hand, the expression of most virulent factors which plays a central role in the organism’s ability to cause diseases [6,7] is regulated by the quorum-dependent accessory gene regulator (agr locus) which expresses two primary divergent transcripts RNAII and RNAIII [6]. The resistance mechanism against methicillin is acquired through the mec gene, which is a part of the staphylococcal cassette chromosome mec [8]. Several MRSA strains also have shown resistance to vancomycin and teicoplanin, and these two antibiotics have often been used to treat MRSA infections [9]. This has been linked to hospital-acquired colonization and the large increase in death and infection rates in nosocomial settings [10].

In addition to the emergence of antibiotic resistance, there has been an increased interest in studying antimicrobial properties of essential oils (EOs) from plant extracts, due to the urgent need for new therapeutic agents. It is rational to expect a selection of plant compounds in these EOs with specific antibacterial activities [11,12]. EOs (known as volatile oils) are aromatic liquid oils obtained from plant materials such as herbs, flowers, buds, leaves, wood, fruits, twigs, bark, seeds, and roots [13,14]. EOs and other plant extracts have antibacterial, antifungal, antiparasitic, antiviral, antioxidant, anti-inflammatory, and anticarcinogenic characteristics and have been screened worldwide as possible sources of new antimicrobial compounds, as novel potential drugs to treat infectious diseases [15-21].

This study was designed to evaluate the effect of several commercially available EOs against clinical isolates of MRSA and to evaluate the inhibitory activity of EOs on the expression level of some virulence genes during the different growth stages of bacteria to test their potential for use as new therapeutic agents.

## Materials and Methods

### Ethical approval

Approval from the Animal Care and Use Committee was not required; this study did not work on humans or animals.

### EOs composition and bacterial strains

This study was conducted in the Microbiology Research Laboratory of the Faculty of Veterinary Medicine, Jordan University of Science and Technology, from January to September 2017. Eight commercial EO blends (A-H) were used to determine their minimum inhibitory concentration (MICs) and MBCs. (1) Six of the EOs formulas (A-F) were supplied by Animal Wellness Products, Reggio Emilia, Italy. The chemical composition of the six EOs is presented in Table-1. (2) Digestarom^®^ P.E.P marked as EO (G) was commercially available from BIOMIN Holding GmbH, Austria. (3) MENTOFIN^®^ marked as EO (H) was commercially available from EWABO Chemikalien GmbH, Germany. All oils were stored at 4°C until used. MRSA strain (ATCC 43300) and clinical isolates of MRSA (marked as 1, 2, 3, D20, D9, 37, and 34) were obtained from Microbiology Laboratory, Department of Basic Medical Veterinary Sciences, Jordan University of Science and Technology.

**Table-1:** Composition (%) of individual EOs.

Composition	EO (A)	EO (B)	EO (C)	EO (D)	EO (E)	EO (F)
b- Pinene	-	-	29.74	-	-	-
Carvacrol	-	62.50	-	39.32	-	46.29
Carvone	-	-	-	37.75	67.14	44.44
Dimethyl trisulfide	17.99	2.99	5.82	-	18.87	-
Linalool	57.60	-	-	-	-	-
Limonene	11.07	-	13.36	-	-	-
Menthol	-	26.24	51.08	-	-	-
Monoterpene hydrocarbons	-	-	-	15.07	-	-
Thymol	13.34	8.27	-	7.87	14.00	9.27

EO=Essential oil

### Antibacterial activity assays

#### Disk diffusion assay

Screening of EOs for antibacterial activity was determined using disk diffusion susceptibility method according to the standard protocols of Clinical and Laboratory Standards Institute (CLSI, 2014), selected MRSA strains were cultured overnight in Mueller-Hinton Broth (MHB) (Oxoid, UK), after that, bacterial cultures were adjusted to McFarland turbidity standard (0.5) with MHB. A sterile cotton swab was immersed in bacterial suspension and was used to streak on the surface of Mueller-Hinton Agar (MHA) plates (Oxoid, UK). Fifteen µL of each EO was impregnated on a sterile blank disc (Whatman disc, 6 mm diameter) (Oxoid, UK). All discs were dried in a laminar flow hood for around 45min before they placed onto the inoculated plates. Then, plates were incubated for 15min at room temperature followed by overnight incubation at 37°C. After 24h of incubation, the inhibition zone diameter was measured in millimeters. All experiments were performed in triplicates.

### MIC and minimum bactericidal concentration (MBC) determination

The inoculum was prepared as described in disk diffusion assay and was then diluted 10-fold to reach a final concentration of 5×10^6^ CFU/ml. The antimicrobial activity against the following MRSA strains was examined: Reference strain of MRSA (ATCC 43300), clinical isolates of MRSA strains (37 and 1). The MIC of all EOs was determined by broth microdilution method using 96-well microtiter plates according to the standard protocols of (CLSI, 2014) with some modification. Briefly, the inoculum was prepared as described above. A2-fold serial dilution of each EO stock (50 µl) in MHB (Oxoid, UK) was prepared in 96-well microplates except the last two columns, which served as negative controls (bacterial inoculum and MHB without EO). Fifty µL of prepared bacterial suspensions (1×10^6^ CFU/ml) were added to each well to reach a final concentration of approximately 5×10^5^ CFU/ml. After 24h of incubation at 37°C, MIC was determined as the lowest concentration of the EO inhibiting visible bacterial growth. The MBC was determined by subculturing 100 µl onto MHA (Oxoid, UK) from wells showing no turbidity next to the MIC well. The MHA was incubated at 37°C for 24h and the lowest concentration without apparent microbial growth was considered as the MBC. Values are the averages of three independent experiments.

### Growth curve and EOs treatment

Growth curves of MRSA used in this study were constructed to determine the time points by which the treatment with the selected oil can be applied. Late exponential phases of growth were best selected due to the stability and non-fragility of the strains [22]. To determine the expression of resistance and virulence genes in different growth phases (different time points) of MRSA (ATCC 43300) without treatment, the growth of the cells was monitored by measuring the OD at 600nm wavelength values at the time points of 2, 4, 6, 8, and 10h. Based on the results of MIC antimicrobial activity against MRSA (ATCC 43300), EOs (A) and (E) were selected. Furthermore, according to the growth curve assay, three time points were determined to harvest the pellets at 2, 5, and 8h. For RNA isolation, overnight cultures of MRSA (ATCC 43300) were inoculated in three flasks 250ml each one containing 150ml MHB II. The cultures were adjusted by spectrophotometer at an initial density OD of 0.05 at 600nm wavelength, and then, 1/3 MIC (V/V) of EO (A) or (E) was added to two flasks (one for EO [A] and the other for EO [E]). After that, cultures were incubated at 37°C with shaking at 160rpm. Cultures with or without EOs were incubated aerobically at 37°C and shacked at 160rpm using shaking incubator and were harvested by centrifugation after 2, 5, and 8h at 4°C. Samples were collected and processed to analyze RNA samples at three time points (2, 5, and 8h) as described in the growth curve assay.

### RNA preparation

#### Total RNA extraction

Bacterial RNA extraction was carried out using an RNeasy^®^ Protect Bacteria Mini Kit (Qiagen, Germany) according to manufacturer’s instructions with minor modification as summarized hereafter: The bacterial cells were pelleted by centrifugation at 4000rpm for 20min. The stabilized cell pellets were resuspended in 1ml of Trizol reagent (Tri Reagent^®^ solution, Thermo Fisher Scientific, USA) and used immediately. To destroy the bacterial cell wall, the suspension was sonicated for 10 s, 4times by ultrasonic probe sonicator (RS, USA) and then vortex vigorously. The suspension was transferred to 1.5ml Eppendorf tube, centrifuged for 1min at 10000rpm, and the supernatant was transferred to the new 1.5ml Eppendorf tube. An equal volume of absolute ethanol was added and mixed with the pipette. Total volume of 700 µl was transferred to an RNeasy Mini Spin Column placed in 2ml collection tube. The rest of the steps follow the kit manufacturer’s instructions. Finally, RNA was eluted by adding 40 µl of RNase-free water and immediately stored at −70°C until further analysis.

### Reverse transcription (RT) reaction

cDNA was synthesized from purified RNA of EO (A) and (E) treated and untreated samples using QuantiTect^®^ Reverse Transcription Kit (Qiagen) according to the manufacturer’s instructions. cDNA was stored directly at −20°C for subsequent real-time polymerase chain reaction (PCR).

### Quantitative real-time PCR (qRT-PCR) reaction

SYBR green dye was used to perform the real-time reaction on a Rotor-Gene Q Cycler^®^ (Qiagen). The primers of genes tested in this study are presented in Table-2. The amplification program was as follows: 95°C for 5min followed by 40cycles of 10 s at 95°C and 30 s at 60°C. The 16S rRNA gene was used as a housekeeping gene. Each cDNA sample of both EOs treated and untreated bacteria was analyzed in triplicates. Fold change in the expression of the genes due to EO treatment was calculated based on ∆∆C_T_ method [23].

**Table-2:** Primers used in this study.

Name	Sequence
mecA	F; 5′-TCCAGATTACAACTTCACCAGG-3′
	R; 5′-CCACTTCATATCTTGTAACG-3′
ica-9	F;5’- TCGCACTCTTTATTGATAGTCGCTACGAG-3’
	R;5’- TGCGACAAGAACTACTGCTGCGTTAAT-3’
ica-15	F;5’- CGACGTTGGCTACTGGGATACTGATATGA-3’
	R;5’- AAATGCGACAAGAACTACTGCTGCGTTAAT-3’
RNAIII	F; 5’-GATGTTGTTTACGATAGCT-3’
	R; 5’-TTCAATGGCACAAGATATC-3’
16S rRNA	F; 5’-CTGTGCACATCTTGACGGTA-3’
	R; 5’-TCAGCGTCAGTTACAGACCA-3’

ica=Intercellular adhesion cluster

### Statistical analysis

All the experimental results were performed in triplicates and the results were expressed as mean±standard deviation for every type of bacterium. Calculations were performed using Microsoft Excel 2016 software.

## Results

### Antibacterial activity of EOs

In the present study, the antimicrobial activity of eight different commercially EO (A-H) blends was screened *in vitro* against clinical MRSA isolates, using the disk diffusion method. The results of this experiment are shown in Table-3. The results revealed that the selected EOs showed varying values of antibacterial activity. In general, most of the tested organisms were sensitive to different types of EOs. Out of eight EOs tested, seven showed antibacterial activity against one or more bacterial strains. EO (A) showed the highest antimicrobial activity against all tested pathogenic MRSA (the range of inhibition zone from 16.7mm to 21mm) followed by EOs (E) (from 12.66mm to 15.00mm) and then (D) (from 11.66mm to 16.00mm). The lowest inhibition zone was observed in EO (C) with inhibition zone range (from 8mm to 12.66mm). In the case of EO (H), there was no observed antimicrobial activity. Values are the averages of three independent experiments.

**Table-3:** Inhibition zone diameter in mm as established by disk diffusion method.

EOs	ATCC 43300	MRSA D9	MRSA D20	MRSA 34	MRSA 37	MRSA 1	MRSA 2	MRSA 3
A	20.33±3.21	16.66±4.50	17.66±2.51	20.66±3.78	21.00±2.64	16.66±1.52	17.33±4.16	17.33±2.51
B	11.66±2.51	10.00±0.00	08.66±1.15	11.00±2.64	15.66±2.88	13.00±2.64	13.00±4.35	10.00±0.00
C	12.33±1.52	08.00±0.00	08.00±0.00	08.33±0.57	08.66±1.15	12.66±5.03	12.00±4.35	09.00±1.00
D	16.00±2.64	13.33±1.15	11.66±1.52	12.66±1.52	13.33±2.51	13.66±0.57	14.66±0.57	12.00±0.00
E	15.00±2.64	14.00±1.00	12.66±1.15	15.00±1.00	13.66±0.57	15.00±1.00	15.00±1.00	15.00±2.64
F	08.66±0.57	10.66±1.15	08.66±0.57	10.66±3.78	13.33±4.16	09.66±0.57	08.33±0.57	10.66±4.61
G	12.66±1.15	11.66±1.15	11.66±0.57	12.33±1.52	11.33±2.3	11.66±0.57	11.00±1.00	09.33±1.52
H	*	*	*	*	*	*	*	*

Values are mean inhibition zone (mm)±SD of three replicates. The diameter of the filter paper disks (6 mm) is included.

* No inhibition zone formation. EO= Essential oil, MRSA=Methicillin-resistant *Staphylococcus aureus*, SD=Standard deviation

### MIC and MBC

The MIC and MBC average of the eight EOs are shown in Tables-4 and 5, respectively. In agreement with the disk diffusion results, most of the EOs showed antimicrobial activity against selected pathogenic bacteria. Among all strains used in this study, reference strain of MRSA (ATCC 43300) was the most susceptible bacteria followed by clinical isolates of MRSA 37 and 1, respectively. For a more detailed of MRSA (ATCC 43300), EOs (E), (D), and (G) have shown the most effective antibacterial activity with MIC (0.4, 0.65, and 0.89 µl/ml) and MBC (1.54, 0.97, and 6.02 µl/ml, respectively) followed by EO (A), (B), (C), and (F), respectively. Finally, EO (H) showed the lowest effective antibacterial activity against MRSA (ATCC 43300) with MIC and MBC of 31.25 µl/ml. In the same order of activity for MRSA (37), EOs (G) and (D) have shown the highest antibacterial activity followed by EOs (A) and (E). While for MRSA (1), EO (G) had shown the highest antibacterial activity followed by EO (A). Most of the EOs had MBC values that were higher than their MIC, which indicates that these EOs are not bactericidal at the MIC.

**Table-4:** MIC (μl/mL) of EOs against three MRSA bacteria using microdilution method.

EOs	ATCC 43300	MRSA 37	MRSA 1
A	3.90±3.38	1.95±1.49	2.76±1.97
B	3.9±0.00	2.60±1.12	4.23±3.42
C	6.51±2.25	6.51±2.25	5.85±3.38
D	0.65±0.28	0.73±0.42	5.69±8.60
E	0.40±0.14	1.38±0.98	6.02±8.34
F	6.51±2.25	2.60±1.12	5.85±3.38
G	0.89±0.92	0.65±0.28	0.40±0.14
H	31.25±0.00	62.5±0.00	16.92±13.71

Values are MIC (μl/ml)±SD of three replicates. EO=Essential oil. MRSA=Methicillin-resistant*Staphylococcus aureus*, MIC=Minimum inhibitory concentration. SD=Standard deviation

**Table-5:** MBC (μl/mL) of EOs against tree MRSA bacteria using microdilution method.

EOs	ATCC 43300	MRSA 37	MRSA 1
A	6.51±7.89	3.90±0.00	4.06±3.66
B	5.20±2.25	20.83±9.02	5.53±3.94
C	9.11±5.96	20.83±9.02	8.46±6.85
D	0.97±0.84	0.73±0.42	5.69±8.6
E	1.54±2.04	1.62±0.56	6.02±8.34
F	9.11±5.96	15.62±0.00	5.85±3.38
G	6.02±8.34	5.85±3.38	0.97±0.84
H	31.25±0.00	62.5±0.00	16.92±13.71

Values are MBC (μl/ml)±SD of three replicates. MBC=Minimum bactericidal concentration, EO=Essential oil, MRSA=Methicillin-resistant *Staphylococcus aureus,* SD=Standard deviation

### Expression levels of virulence genes quantified by qRT-PCR

**Figure-1:**
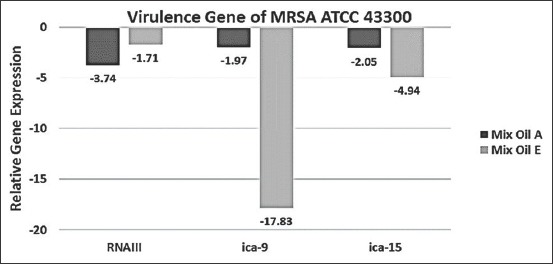
Relative expression of RNAIII, intercellular adhesion cluster (ica)-9, and ica-15 in *Staphylococcus aureus* (ATCC 43300) was cultured with 1/3minimum inhibitory concentration of essential oils (A) and (E) to the post-exponential growth phase (t=8h). Transcript levels were monitored by quantitative real-time polymerase chain reaction as described in the Materials and Methods. The relative gene expression levels were normalized to 16S rRNA, and the drug-free culture was used as a calibrator. Value >0 indicates upregulation and <0 indicates down-regulation.

The qRT-PCR was performed only in samples collected after 8h, because the pellet size after 2 and 5h was too small to perform the RNA extraction. The relative threshold cycle (C_T_) method was used to analyze the results. The expressions of examined virulence gene were significantly down-regulated in samples treated with EO (E) compared to control. These results indicate that intercellular adhesion genes ica-9 and ica-15 were down-regulated by 17.83 and 4.94 folds, respectively. The relative expression levels of RNAIII in sample treated with EO (E) showed down-regulation by 1.71 folds. Similarly, the level of intercellular adhesion gene ica-9, intercellular adhesion gene ica-15, and RNAIII was decreased by 1.97-2.05-3.74 folds, respectively, in samples treated with EO (A) (Figure-1).

## Discussion

With increasing resistance of microorganisms to the currently used antibiotic drugs and the high cost of production of new synthetic compounds, pharmaceutical companies are now looking for antibiotics alternatives. Medicinal plants could be a good alternative because most of them are safe with little side effects and less cost and affect a wide range of antibiotic-resistant microorganisms. Within all eight tested EO blends, there was a variation in their antibacterial activity against most tested bacteria, and this variation could be related to the differences in their contents of active ingredients, which are different from blend to other, that is, mean each EO contains components that may not exist in the other one. Indeed, several factors such as temperature, inoculum size, strain, and test methods could change the MIC values. In addition, it is difficult to monitor the rate of solubility of natural EOs [24]. Based on that, an attempt was made to keep all the experimental conditions identical to compare the results of our study. Interestingly, among all MRSA strains used in this study, reference strain of MRSA (ATCC 43300) was found as the most susceptible bacteria, which being sensitive to all assayed EOs. According to the literature, EOs contained many ingredients and their antimicrobial activity cannot be certain depending only on the action of one component [25]. The ability of *S. aureus* to cause a variety of serious infections in humans is linked to the expression of a group of agents that cooperate in the pathogenesis of infection in humans or animals[26]. These agents lead to adhesion of this bacterium to the surfaces of host tissue, invasion or avoidance of the immune system, and causing damage in the host by the effects of its toxins [27,28]. These agents are known as virulence factors and are divided to adherence or exotoxin factors. Therefore, clinically, performance of antimicrobial drugs used in the treatment of *S. aureus* infections not only depends on the respective bacteriostatic or bactericidal nature of the antibiotic but also on the alternate strategy that targets bacterial virulence factors (e.g.enterotoxins, hemolysins, and adhesins) [29]. Several studies have investigated the changes in gene expression patterns that exhibit little or no influence in response to antibiotics at the sub-MIC concentrations. Ohlsen *et al*. found that sub-MIC of various antibiotics modulates the expression of the hla gene, encoding staphylococcal alpha-toxin (hla) [30]. Another study by Koszczol *et al*. [31] suggested that sub-MIC quinupristin/dalfopristin inhibits virulence factors release by *S. aureus* (e.g.autolysin, protein A, and b-hemolysins, lipases). The effect of antibiotics on regulation of virulence factors may result in either disturbance or attenuation of the infection. Indeed, plant EOs have a multicomponent nature, it is more difficult for bacteria to develop resistance than many common used antibiotics, which have a single target site [32,33]. Many studies demonstrated that some plant EOs (e.g.,oils of cinnamon, bay, and clove) can suppress the production of virulence factors when used at sub-MIC concentrations [32]. For example, sub-MIC concentrations of thymol plant decreased exotoxin production in *S. aureus*, possibly in part due to inhibition of the agr locus [34]. The expression of most *S. aureus* virulence factors is regulated by a network of interacting regulators, such as agr, staphylococcal accessory regulator A, and staphylococcal accessory element (sae) [29]. The previous studies have shown that sub-MIC concentrations of antibiotics can affect the translation of certain regulatory gene products in *S. aureus*, which, in turn, alter the transcription of toxin-encoding genes. The pathogenicity of *S. aureus* is a complicated process that involves a various set of cell wall and extracellular components working in coordination over several stages of infection [26]. The systems of *S. aureus* (two-component regulatory) involve agr[35] and sae [36]. The agr locus regulates the expression of the gene coding for small RNA, recognized as RNA III, also known as hld, and encodes for delta-hemolysin. The RNAIII is an effector molecule of agr locus and it works as sensor to the agr locus in response to environmental conditions [37]. In addition, the agr locus regulates the expression of many virulence factors. For example, it is responsible for down-regulation of cell wall-associated proteins synthesis such as FnbpA, FnbpB, and SpA and upregulation of the expression of several exoproteins such as α-hemolysin, serine proteinase, toxic shock syndrome toxin-1, enterotoxins, and proteases [38,39]. The sae locus codes are responsible for regulating the expression of several virulence factors including bacterial adhesion, toxicity, and immune evasion [40]. This includes the upregulation of α-, β-, and γ-hemolysins [41] and the down-regulation of SpA [42]. In this study, quantitative RT-PCR was used to investigate the influence of EOs on the RNAIII which is a part of the agr locus and at the same time, it is responsible for activating it in *S. aureus*. The results of this study detected a reduction in expression of RNAIII when *S. aureus* strain (ATCC 43300) was cultured with 1/3 MIC of EO (A) by 3.74 fold and (E) by 1.71 fold. Therefore, we believe that the reduction in the production of agr locus, in part, depends on the inhibition of the RNAIII induced by EOs (A) and (E) that might lead to suppress or reduce the effect of *S. aureus* virulence factors. The ica ADBC operon was first identified in *S. epidermidis* and then in *S. aureus*. The ica locus is a portion of the “accessory genes” of bacterial genome, but not present in all bacterial strains. The ica gene plays an important role in biofilm formation in response to stress factors, a developmental process that requires polysaccharide intercellular adhesion [5]. Absent of the ica locus results in an incapacity to produce polysaccharide intercellular adhesin and leads to prevent biofilms formation [5]. This biofilm makes the bacteria more resistant to antibiotics. Interestingly, our results indicated that the expression level of ica-9 and ica-15 was significantly down-regulated when MRSA (ATCC 43300) was cultured with 1/3 MIC of EO (E) by 17.83 of ica-9 and 4.94 fold of ica-15. While, the ica-9 and ica-15 expression levels were less affected by EO (A) with 1.97 fold of ica-9 and 2.05 fold of ica-15. In agreement with our results, Yadav *et al*. [43] demonstrated that ica gene of MRSA was significantly down-regulated by 1.3, 6.1, and 3.4 folds at 24, 36, and 48h post-treatment of eugenol oil. Therefore, we believe that EO (E) interferes in the expression of ica genes that are related to biofilm formation. This leads to suppression of the virulence factors of MRSA (ATCC 43300) and, therefore, decreases its pathogenicity and resistance to treatment of the infected host. The efficacy of EOs against the MRSA in this study has yet to be confirmed through further researches for their role in MRSA biofilm eradication and to conduct further *in vivo* trials.

## Conclusion

These encouraging results indicate that these EOs can be utilized as a natural antibiotic for the treatment of MRSA disease.

## Authors’ Contributions

MHG and MSK: Conceptualization. MHG and MSK: Data curation. MHG, MSK, EMZ, and LFA: Formal analysis. MHG, EMZ, and LFA: Methodology. MHG, MSK, and LFA: Project administration. MHG and MSK: Supervision. MHG and EMZ: Writing of original draft, MHG and LFA: Writing, review and editing. All authors read and approved the final manuscript.

## References

[1] Khan H.A, Ahmad A, Mehboob R (2015). Nosocomial infections and their control strategies. Asian Pac. J. Trop. Biomed.

[2] Barber M (1961). Methicillin-resistant staphylococci. J. Clin. Pathol.

[3] Lee D.S, Kang M.S, Hwang H.J, Eom S.H, Yang J.Y, Lee M.S, Lee W.J, Jeon Y.J, Choi J.S, Kim Y.M (2008). Synergistic effect between dieckol from *Ecklonia stolonifera* and β-lactams against methicillin-resistant *Staphylococcus aureus*. Biotechnol. Bioprocess Eng.

[4] Eom S.H, Kim D.H, Lee S.H, Yoon N.Y, Kim J.H, Kim T.H, Chung Y.H, Kim S.B, Kim Y.M, Kim H.W, Lee M.S, Kim Y.M (2013). *In vitro* antibacterial activity and synergistic antibiotic effects of phlorotannins isolated from *Eisenia bicyclis* against methicillin-resistant *Staphylococcus aureus*. Phytother. Res.

[5] Ghasemian A, Peerayeh S.N, Bakhshi B, Mirzaee M (2015). High prevalence of icaadbc genes responsible for biofilm formation in clinical isolates of *Staphylococcus aureus* from hospitalized children. Arch. Pediatr. Infect. Dis.

[6] Richard P.N (2000). Pathogenicity Factors and Their Regulation. Gram-Positive Pathogens.

[7] Paharik A.E, Horswill A.R (2016). The *Staphylococcal* biofilm:Adhesins, regulation, and host response. Microbiol. Spectr.

[8] Blair J.M.A, Webber M.A, Baylay A.J, Ogbolu D.O, Piddock L.J.V (2015). Molecular mechanisms of antibiotic resistance. Nat. Rev. Microbiol.

[9] Bal A.M, David M.Z, Garau J, Gottlieb T, Mazzei T, Scaglione F, Tattevin P, Gould I.M (2017). Future trends in the treatment of methicillin-resistant *Staphylococcus aureus*(MRSA) infection:An in-depth review of newer antibiotics active against an enduring pathogen. J. Glob. Antimicrob. Resist.

[10] Witte W, Braulke C, Cuny C, Strommenger B, Werner G, Heuck D, Jappe U, Wendt C, Linde H.J, Harmsen D (2005). Emergence of methicillin-resistant *Staphylococcus aureus* with panton-valentine leukocidin genes in central Europe. Eur. J. Clin. Microbiol. Infect. Dis.

[11] Gogoi R, Loying R, Sarma N, Munda S, Pandey S.K, Lal M (2018). A comparative study on antioxidant, anti-inflammatory, genotoxicity, anti-microbial activities and chemical composition of fruit and leaf essential oils of *Litsea cubeba* Pers from North East India. Ind. Crops Prod.

[12] Brochot A, Guilbot A, Haddioui L, Roques C (2017). Antibacterial, antifungal, and antiviral effects of three essential oil blends. Microbiologyopen.

[13] Devi K.P, Nisha S.A, Sakthivel R, Pandian S.K (2010). Eugenol (an essential oil of clove) acts as an antibacterial agent against *Salmonella* typhi by disrupting the cellular membrane. J. Ethnopharmacol.

[14] Lorenzi V, Muselli A, Bernardini A.F, Berti L, Pagès J.M, Amaral L, Bolla J.M (2009). Geraniol restores antibiotic activities against multidrug-resistant isolates from gram-negative species. Antimicrob. Agents Chemother.

[15] Alviano D.S, Alviano C.S (2009). Plant extracts:Search for new alternatives to treat microbial diseases. Curr. Pharm. Biotechnol.

[16] Gadisa E, Weldearegay G, Desta K, Tsegaye G, Hailu S, Jote K, Takele A (2019). Combined antibacterial effect of essential oils from three most commonly used Ethiopian traditional medicinal plants on multidrug-resistant bacteria. BMC Complement. Altern. Med.

[17] Kang J, Jin W, Wang J, Sun Y, Wu X, Liu L (2019). Antibacterial and anti-biofilm activities of peppermint essential oil against *Staphylococcus aureus*. LWT.

[18] Giarratana F, Muscolino D, Ziino G, Lo Presti V, Rao R, Chiofalo V, Giuffrida A, Panebianco A (2017). Activity of catmint (*Nepeta cataria*) essential oil against anisakis larvae. Trop. Biomed.

[19] Hirota R, Roger N.N, Nakamura H, Song H.S, Sawamura M, Suganuma N (2010). Anti-inflammatory effects of limonene from yuzu (*Citrus junos* Tanaka) essential oil on eosinophils. J. Food Sci.

[20] Shahbazi Y (2017). Chemical compositions, antioxidant and antimicrobial properties of *Ziziphora clinopodioides* Lam. Essential oils collected from different parts of Iran. J. Food Sci. Technol.

[21] Lu X.G, Bin Z.L, Feng B.A, Qu M.Y, Yu L.H, Xie J.H (2004). Inhibition of growth and metastasis of human gastric cancer implanted in nude mice by d-limonene. World J. Gastroenterol.

[22] Abu-Qatouseh L.F, Chinni S.V, Seggewiß J, Proctor R.A, Brosius J, Rozhdestvensky T.S, Peters G, von Eiff C, Becker K (2010). Identification of differentially expressed small non-protein-coding RNAs in *Staphylococcus aureus* displaying both the normal and the small-colony variant phenotype. J. Mol. Med.

[23] Schmittgen T.D, Livak K.J (2008). Analyzing real-time PCR data by the comparative C(T) method. Nat. Protoc.

[24] Ye H, Shen S, Xu J, Lin S, Yuan Y, Jones G.S (2013). Synergistic interactions of cinnamaldehyde in combination with carvacrol against food-borne bacteria. Food Control.

[25] Chouhan S, Sharma K, Guleria S (2017). Antimicrobial activity of some essential oils-present status and future perspectives. Medicines (*Basel*).

[26] Oliveira D, Borges A, Simões M (2018). *Staphylococcus aureus* toxins and their molecular activity in infectious diseases. Toxins.

[27] Dinges M.M, Orwin P.M, Schlievert P.M (2000). Exotoxins of *Staphylococcus aureus*. Clin. Microbiol. Rev.

[28] Majerczyk C.D, Sadykov M.R, Luong T.T, Lee C, Somerville G.A, Sonenshein A.L (2008). *Staphylococcus aureus* CodY negatively regulates virulence gene expression. J. Bacteriol.

[29] Kane T.L, Carothers K.E, Lee S.W (2018). Virulence factor targeting of the bacterial pathogen *Staphylococcus aureus* for vaccine and therapeutics. Curr. Drug Targets.

[30] Ohlsen K, Ziebuhr W, Koller K.P, Hell W, Wichelhaus T.A, Hacker J (1998). Effects of subinhibitory concentrations of antibiotics on alpha-toxin (hla) gene expression of methicillin-sensitive and methicillin-resistant *Staphylococcus aureus* isolates. Antimicrob. Agents Chemother.

[31] Koszczol C, Bernardo K, Krönke M, Krut O (2006). Subinhibitory quinupristin/dalfopristin attenuates virulence of *Staphylococcus aureus*. J. Antimicrob. Chemother.

[32] Smith-Palmer A, Stewart J, Fyfe L (2004). Influence of subinhibitory concentrations of plant essential oils on the production of enterotoxins A and B alpha-toxin by *Staphylococcus aureus*. J. Med. Microbiol.

[33] García-Salinas S, Elizondo-Castillo H, Arruebo M, Mendoza G, Irusta S (2018). Evaluation of the antimicrobial activity and cytotoxicity of different components of natural origin present in essential oils. Molecules.

[34] Qiu J, Wang D, Xiang H, Feng H, Jiang Y, Xia L, Dong J, Lu J, Yu L, Deng X (2010). Subinhibitory concentrations of thymol reduce enterotoxins A and B and alpha-hemolysin production in *Staphylococcus aureus* isolates. PLoS One.

[35] Peng H.L, Novick R.P, Kreiswirth B, Kornblum J, Schlievert P.M (1988). Cloning, characterization, and sequencing of an accessory gene regulator (agr) in *Staphylococcus aureus*. J. Bacteriol.

[36] Giraudo A.T, Raspanti C.G, Calzolari A, Nagel R (1994). Characterization of a Tn 551-mutant of Staphylococcus aureus defective in the production of several exoproteins. Can. J. Microbiol.

[37] Novick R.P, Geisinger E (2008). Quorum sensing in staphylococci. Annu. Rev. Genet.

[38] Novick R.P, Ross H.F, Projan S.J, Kornblum J, Kreiswirth B, Moghazeh S (1993). Synthesis of staphylococcal virulence factors is controlled by a regulatory RNA molecule. EMBO J.

[39] Oscarsson J, Tegmark-Wisell K, Arvidson S (2006). Coordinated and differential control of aureolysin (aur) and serine protease (sspA) transcription in *Staphylococcus aureus* by sarA, rot and agr (RNAIII). Int. J. Med. Microbiol.

[40] Rogasch K, Rühmling V, Pané-Farré J, Höper D, Weinberg C, Fuchs S, Schmudde M, Bröker B.M, Wolz C, Hecker M, Engelmann S (2006). Influence of the two-component system SaeRS on global gene expression in two different *Staphylococcus aureus* strains. J. Bacteriol.

[41] Goerke C, Fluckiger U, Steinhuber A, Bisanzio V, Ulrich M, Bischoff M, Patti J.M, Wolz C (2005). Role of *Staphylococcus aureus* global regulators sae and sigmaB in virulence gene expression during device-related infection. Infect. Immun.

[42] Giraudo A.T, Cheung A.L, Nagel R (1997). The sae locus of *Staphylococcus aureus* controls exoprotein synthesis at the transcriptional level. Arch. Microbiol.

[43] Yadav M.K, Chae S.W, Im G.J, Chung J.W, Song J.J (2015). Eugenol:A phyto-compound effective against methicillin-resistant and methicillin-sensitive *Staphylococcus aureus* clinical strain biofilms. PLoS One.

